# Letter from Italy

**Published:** 1885-02

**Authors:** A. Lagorio

**Affiliations:** Chiavari


### FOREIGN CORRESPONDENCE.

LETTER FROM ITALY.

*Messrs. Editors*—During this century no remedy has attracted such great attention as cocaine. Its remarkable properties have been praised by every medical man in all the countries of the world. All the medical journals are enthusiastic in their reports, and the study of this alkaloid is the most important question of the day. At the risk of being a repeater, I have availed myself of a very interesting review by Prof. Rummo (*Riforma Medica*), to present to your readers what is known regarding cocaine and its effects. As soon as Charles Koller, of the Hospital of Vienna, communicated, at the Ophthalmological Congress of Heidelberg, his experiments made in the laboratory of Stricker, regarding the anæsthethic action of cocaine on the cornea and conjunctiva, every ophthalmologist and the most esteemed experimenters of all nations procured the remedy, and with a febrile activity they studied its physiological action and therapeutical properties. They are all unite to affirm that the property of cocaine, to render absolutely insensible the cornea, marks a discovery of the highest importance in the ocular therapy.

Cocaine ($C_{17}$ $H_{21}$ Az $O_1$) is a crystallized alkaloid, extracted by Niemann in 1859 from the leaves of coca (erythroxylon coca), a plant of South America. The annual consumption of this plant is over fifteen millions of kilograms, which represent a sum of about eight millions of dollars. Cocaine crystallizes in small colorless prisms, slightly soluble in water, soluble in alcohol, and most soluble in ether; has a slight bitter taste, and a reaction strongly alkaline. The muriate is the salt most frequently used. This salt is soluble in water in the proportion of 5 per cent. without the addition of any acid; but the solution remains opalescent, becoming clear by filtration. Merck has also prepared the salicylate and the bromhydrate of cocaine.

Before the discovery made by Koller, several authors, as Woehler, Rossier, Demarle, Schroff, Loessen, Lippmann, Moreno y Maiz and recently Von Aurep (1880) and Sigmund Freud (*Centralblatt f. Therapie v. Heitler, 1884*) had already studied the physiological and chemical action of cocaine, but no one had spoken regarding its anæsthetic action on the cornea. The experiments of Schroff had shown that the injection of five to ten centigr. of cocaine in the subcutaneous connective tissue of the rabbit produced tetanic and epileptiform convulsions, great dilatations of the pupils, increased frequency of the respiratory acts and some cardiac contractions. It was also known that cocaine produced a general anæsthesia, and when placed in contact with the tongue it caused a complete anæsthesia. Cocaine was used by Coupard and Fauvel in 1870 and 1880 as an anæsthetic substance of the nasal mucous membrane. These effects inspired the idea in Koller to try cocaine as an anæsthetic of the cornea and conjunctiva. He made his first experiments in animals, and by dropping a solution of muriate of cocaine in the eye, he was enabled to produce a complete insensibility of the cornea and conjunctiva; a similar result he obtained in an inflamed cornea (traumatic keratitis). The instillation of cocaine in the eye produced also a diminution of the clearness in the vision of the objects, a paresis ot accommodation, and a dilatation of the pupil. The mydriasis reaches the highest grade in the course of the first hour, diminishes during the second to gradually disappear in the successive hours. Koller reached these conclusions: 1st, the anæsthetic action of cocaine is cumulative; that is, if the remedy is instilled during the moment in which the first anæsthesia disappears, there is obtained a second insensibility of greater duration than the first; 2d, the action of cocaine is essentially local; that is, it acts more powerfully in the point of application and in its surrounding parts.

In the ophthalmological clinic of Prof. Reuss, Koller tried the action of cocaine with two objects: as a narcotic in the painful ocular diseases, as an anaesthetic in ocular surgery. As a *narcotic* he obtained favorable results, especially in the corneal and conjunctival affections, in case of iritis accompanied with photophobia and pains. It is to be noted that the pains and the photophobia returned two or three hours afterwards, and that therefore the remedy is to be periodically applied. Cocaine had no curative property in these affections. Again, the pain produced by the cauterization of the lids with the nitrate of silver is notably diminished, sometimes totally, by a previous instillation of cocaine.

Cocaine possesses a greatest importance as an *anæsthetic* in the surgery of the eye. By its use many operations can be performed on the eye, such as the extraction of foreign bodies from the cornea, without the least resentment on the part of the patients, operations of pterygium, cauterization of ulcers of the cornea, iridectomy, puncture of the cornea and the operation of cataract.

The method of Koller in the clinic of Reuss for the dropping of cocaine in the eye before the operation, is the following: half an hour before the operation, drop, every five minutes, a solution of five per cent. (two drops at a time). The instillation is practiced while the patient lies horizontally on the bed.

Vulpian, surprised by these marvellous results of Koller, and desirous of studying the general action of cocaine, made several experiments which he communicated to the Academy ot Sciences of Paris. He found that the anæsthetic action produced by the general action of cocaine is inferior to the one obtained by a local way; by injecting the remedy in the veins there is produced a dilatation of the pupils and a certain degree of exophthalmos; these phenomena are analogous to those produced by the electrifying of the cervical column of the great sympathetic. The anæsthetic action of cocaine in the mollusks is not decisive. The toxic effects are clearly manifested, and the insensibility is produced in the crustaceaus.

F. Frank communicated to the Biological Society (Nov. 29, 1884) his conclusions on cocaine: 1st, cocaine, which suppresses the sensibility to pain and to the touch, does not suppress the cortical excitability; 2d, cocaine suppresses the excitability of the dura mater; 3d, cocaine has a transitory effect and does not determine any phenomena of local irritations.

Laborde has noticed that cocaine, injected in the subcutaneous connective tissue or in the veins of warm-blooded animals, in small doses, produces agitation or restlessness and analgesia of the mucous membranes of the nose, pharynx, tongue, etc.; in high doses, tetanic and epileptiform convulsions.

Arthur Beeson, of Dublin (*Lancet, Oct. 1884*), Marcus Gunn, Koenigtein, Hock, etc., have confirmed the physiological and therapeutical experiments of Koller.

In France the question was studied by eminent oculists with the greatest interest. Among these are Panas, Abadie, Landolt, Vacher of Orleans, etc. Panas, in one of his communications to the Academy of Medicine of Paris, confirmed, in a great part, the results of Koller, and said that the fugitive mydriatic action of cocaine could be utilized for the ophthalmoscopical exploration of the fundus of the eye, and that in the deep operations of the eye the patient notices only a slight pain in the excision of the iris. He also noted that the inflamed eye is more or less refractory to the anæsthetic action of cocaine.

Abadie (*Bull. gen. de Therap., 15th Nov., 1884*) could not notice any paresis of accommodation, as all patients could read the finest characters at a normal distance. Moreover, he noticed the great benefits of cocaine in the operations and in the painful affections of the eye, excepting the section of the iris and the extirpation of the eye, in which the patients frequently feel the greatest pains notwithstanding the previous application of cocaine. Landolt, (*Archiv. d'Opht., No. 5, 1884*) in the deep operations of the eye, attempted to instill the cocaine in a button-hole incision, made in the conjunctiva. When the remedy is injected in the lachrymal ducts, it renders more tolerable the passage of the sound.

Vacher (*Gaz. Hebd., No. 48, 1884*), in ocular operations, has practiced both the direct instillation and the subcutaneous injections on the course of the sensitive nerves of the region, and by so doing he observed that cocaine has an action more profound and more lasting.

But the generic physicians could not remain idle after noticing this great progress obtained by the oculists with the use of cocaine, and have tried the effect of cocaine in the affections of the different organs of the body. So far, we know that this remedy has shown its anæsthetic action on the oral, pharyngeal, laryngeal, nasal, urethral and rectal mucous membranes.

Dujardin-Beaumetz has calmed the violent gastric pains with this remedy. He sustains that cocaine can be used with benefit in the morphiomaniacs. Injected under the skin, it produces effects analogous to those of morphine, without the grave inconveniences of this alkaloid. Vacher has used cocaine in the extirpation of teeth, by wetting a piece of cotton with a 0.1 solution and by introducing it in the bottom of the carious tooth. The extirpation, made after ten minutes, seemed less painful.

We can readily see that cocaine is destined to occupy an important position in therapeutics. So far, the best results have been obtained in the ocular surgery, but we may affirm that the muriate of cocaine is destined to substitute chloroform and atropine.

The price of this alkaloid at present is very high, but will gradually decrease as its consumption becomes greater.

A. LAGORIO, M. D.

Chiavari, December 31st, 1884.

